# Spatial–Spectral Bidirectional-Driven Collaborative Network with Coordinate-Aware and Spectral-Modulated Interaction for Hyperspectral Pansharpening

**DOI:** 10.3390/s26103009

**Published:** 2026-05-10

**Authors:** Qingshan Gao, Conghui Tao, Xiongjun Du, Yanmin Zhu, Shixuan Liu, Xiuxiu Chen, Qiuxiao Chen

**Affiliations:** 1 Siwei SuperView Satellite Remote Sensing Co., Ltd., Hangzhou 310012, China; gaoqingshan@swgjsatellite.com (Q.G.); duxiongjun@swgjsatellite.com (X.D.); zhuyanmin@swgjsatellite.com (Y.Z.); 2 Eco-Environmental Science Research & Design Institute of Zhejiang Province, Hangzhou 310007, China; liushixuan@westlake.edu.cn; 3 State Environmental Protection Engineering (Zhejiang) Center for Water Pollution Control, Hangzhou 310007, China; 4 Key Laboratory of Environmental Pollution Control Technology of Zhejiang Province, Hangzhou 310007, China; 5 School of Spatial Planning and Design, Hangzhou City University, Hangzhou 310015, China; cxxtribal@zju.edu.cn (X.C.); chenqx@hzcu.edu.cn (Q.C.)

**Keywords:** hyperspectral pansharpening, bidirectional driving framework, real-world dataset

## Abstract

High-resolution hyperspectral computational imaging is critical for applications such as environmental monitoring, urban planning, and precision agriculture. In practical hyperspectral imaging systems, physical hardware constraints inevitably lead to coupled degradations across spatial and spectral dimensions, making it difficult to simultaneously achieve high spatial resolution and high spectral fidelity. As a representative and widely studied hyperspectral computational imaging task, hyperspectral pansharpening aims to reconstruct high-resolution hyperspectral images by integrating low-resolution hyperspectral images with high-resolution panchromatic images. Existing methods frequently suffer from spectral distortion or blurred spatial details due to unidirectional fusion strategies or isolated processing branches that inadequately model the intrinsic spatial–spectral coupling in the imaging process. To overcome these limitations, we propose a bidirectional driving framework that enables synergistic mutual guidance between spatial detail infusion and spectral fidelity preservation. Specifically, spatial coordinate-aware representations are dynamically integrated into a spectral self-attention module, while spectral importance scores are utilized to modulate multi-receptive-field convolutions via channel-wise weighting. This bidirectional interaction mechanism forms a closed-loop coupling between spatial and spectral representations, ensuring enhanced spatial reconstruction while rigorously preserving spectral integrity. Furthermore, to bridge the gap between simulated experiments and real-world applications, we constructed a large-scale dataset derived from the ZY-1-02D satellite. This dataset features high-fidelity PAN (17,820 × 16,128) and HSI (1485 × 1344) pairs, which we have made publicly available to the community to facilitate future research. Extensive experiments on both benchmark simulations and the proposed ZY-1-02D dataset demonstrate that our method achieves state-of-the-art performance in both spatial fidelity and spectral preservation.

## 1. Introduction

High-resolution hyperspectral computational imaging plays a pivotal role in Earth observation applications such as environmental monitoring [1], urban planning [2], and precision agriculture [3]. By jointly modeling the image acquisition process and post-capture reconstruction, hyperspectral computational imaging seeks to overcome the inherent limitations of optical sensors through algorithmic inference. Dense spectral sampling provides distinctive spectral “fingerprints” for material identification, while high spatial resolution ensures accurate localization of ground objects. However, due to fundamental physical constraints—most notably the trade-off between signal-to-noise ratio, spatial sampling density, and spectral resolution—spaceborne imaging systems are unable to acquire high spatial and spectral resolutions simultaneously.

As a representative and widely adopted hyperspectral computational imaging [4] paradigm, hyperspectral pansharpening aims to alleviate this hardware bottleneck through post-acquisition reconstruction [5]. By fusing a low-resolution hyperspectral image (LR-HSI) with a high-resolution panchromatic image (HR-PAN), pansharpening methods seek to synthesize a high-resolution hyperspectral (HR-HSI) product that preserves both fine spatial structures and faithful spectral signatures [6]. In this sense, pansharpening can be interpreted as a spatial–spectral reconstruction problem driven by complementary observations under heterogeneous sensing conditions.

Despite significant advancements, existing pansharpening methods often struggle to maintain a balance between spatial and spectral fidelity. Most traditional and deep learning-based approaches focus disproportionately on either spatial textural enhancement [7] or spectral preservation [8]. This unidirectional focus frequently results in a “seesaw effect,” where sharper spatial boundaries are achieved at the cost of spectral distortion, or vice versa. To address this, recent research has explored dual-path architectures. However, a major limitation of conventional dual-branch frameworks [9] is their tendency to treat spatial and spectral pathways as isolated modules. Such separation leads to feature imbalance and misalignment, where late-stage fusion (e.g., simple concatenation or addition) fails to resolve the intrinsic redundancy and complex interplay between dimensions. While cross-attention mechanisms [10,11] have been introduced to mitigate this, they are often computationally prohibitive and typically restricted to spectral-wise attention, leaving spatial–spectral cross-interactions underutilized. In addition, some recent studies [12] attempt to model spatial–spectral relationships in a more unified manner by progressively integrating spectral information into spatial representations, enabling closer coupling between the two domains.

To overcome these deficiencies, we propose a bidirectional driving framework that establishes a reciprocal guidance loop between spatial and spectral branches. Unlike traditional methods that rely on passive fusion, our approach implements a dynamic interaction scheme. In contrast to progressive integration strategies, the proposed framework facilitates continuous interaction between spatial and spectral features during feature learning. Specifically, we introduce a spatial coordinate-aware injection strategy within the spectral self-attention module, where learnable positional representations are integrated into spectral tokens to emphasize spatially salient regions. Concurrently, we design a spectral-modulated spatial convolution unit that utilizes spectral importance scores to adaptively weight multi-receptive-field kernels. This reciprocal design forms a coupled interaction mechanism, where spatial context refines spectral features through coordinate-aware attention, while spectral statistics simultaneously guide spatial feature extraction via adaptive channel weighting. Ultimately, this leads to improved reconstruction fidelity.

A critical bottleneck hindering the progress of hyperspectral pansharpening is the scarcity of large-scale, high-quality real-world datasets for training and validation. Most existing studies rely on simulated data, which often fails to capture the complex noise distributions and registration errors present in orbital sensors. To bridge this gap, we constructed and released a new benchmark dataset derived from the ZY-1-02D satellite. This dataset comprises a vast real-world scene with a PAN resolution of 17,820 × 16,128 and a corresponding HSI resolution of 1485 × 1344. By providing such high-fidelity, original-scale data, we offer a more rigorous evaluation platform that reflects true remote sensing challenges.

The main contributions of this work are summarized as follows:

(1) We propose a bidirectional driving framework that enables synergistic mutual guidance between spatial and spectral features, effectively suppressing spectral distortion while enhancing spatial sharpness.
(2) A coordinate-weighted spatial injection and a channel-weighted spectral modulation mechanism, creating a tightly coupled loop for adaptive spatial–spectral interaction.
(3) A large-scale real-world pansharpening dataset from the ZY-1-02D satellite, providing the community with a high-resolution benchmark to bridge the gap between simulation and practical application.
(4) Extensive experiments on both benchmark simulated datasets and the proposed ZY-1-02D real-world data demonstrate that our method consistently achieves superior performance over state-of-the-art approaches, achieving PSNR improvements of approximately 0.5–0.7 dB on WorldView-3, 0.5–0.6 dB on Chikusei, and over 1.4 dB on ZY-1-02D, while maintaining a favorable balance between reconstruction accuracy and computational efficiency.

## 2. Related Works

Traditional methods, such as component substitution [13,14] and multiresolution analysis (MRA) [15], often suffered from spectral distortions and limited spatial detail preservation. With the emergence of deep learning, significant progress has been made in balancing spatial enhancement and spectral fidelity.

Initial deep learning-based methods adapted super-resolution networks originally designed for RGB imagery. SRCNN [16] and its extensions [17] introduced convolutional learning into image reconstruction, and were later applied to pansharpening tasks [18] where stacked LR-MS and PAN images were used as input. This concept was extended to 3D convolutions to accommodate the spectral dimension of HS data, while residual learning was incorporated to emphasize spatial detail enhancement. Building on this foundation, FusionNet [19] adopted the difference between PAN and interpolated MS images as a learning target, inspired by MRA.

More recent efforts introduced dual-branch architectures and attention mechanisms to improve spectral–spatial feature extraction. For instance, DBDENet [20] designed bidirectional branches for spatial detail estimation from HS and PAN data at multiple levels, while S2DMDN [21] separately optimized spatial and spectral branches before fusing them. HyperPNN [22] emphasized spectral prediction by tailoring convolutional blocks to spectral behavior. In parallel, deep multiscale frameworks such as those proposed by Fu et al. [23] captured finer granularity across resolutions using detail-aware modules. To refine this further, models like RHDN [24] merged shallow and deep features to enhance missing details both within and beyond the visible spectrum.

To address spectral–spatial inconsistency, attention mechanisms became central. Several networks, including those with channel–spatial attention modules [25,26,27], modulated features across dimensions for better localization and spectral alignment. Others, like TDNet [28], incorporated hierarchical domain-specific constraints to progressively inject spatial features. Generative models such as PS-GDANet [9] introduced adversarial learning to maintain the balance between spectral integrity and detail enhancement. Transformer-based architectures [29] also emerged, with Spectral–Spatial Transformer [30,31] and HyperTransformer [10] capturing long-range dependencies across spatial and spectral dimensions. Beyond task-specific designs, recent studies have explored more general and scalable representation paradigms. For instance, HyperSIGMA [32] adopts a foundation-model-style transformer with sparse sampling attention, enabling efficient modeling of spectral–spatial redundancy and promoting transferable feature representations across different data distributions.

In parallel, recent works have investigated more flexible representation learning and holistic modeling strategies. For example, ITER [33] introduces an image-to-pixel representation paradigm that bridges coarse-grained supervision and fine-grained feature learning through spectral–spatial activation and alignment mechanisms, highlighting the potential of leveraging weak structural priors for dense representation refinement. Furthermore, the concept of complete modeling has been explored to alleviate the fragmentation of conventional pipelines. The hyperspectral full model [34] emphasizes comprehensive feature exploration, feature reuse, and multidomain differential fusion within a unified framework, providing a more systematic perspective on how heterogeneous features should be jointly modeled and integrated.

Some methods sought to resolve the limitations of independently processing spatial and spectral paths. Although cross-attention-based approaches attempted to fuse both dimensions, spatial attention was often constrained to mitigate computational overhead. MDANet [35], PMACNet [36] and similar models used multi-stage attention mechanisms to fuse informative features and refine reconstruction in complex scenarios. Despite these advancements, challenges remain in effectively integrating spatial and spectral information without introducing redundancies or distortions. Moreover, existing methods still exhibit limitations in unified modeling of heterogeneous features and efficient exploitation of cross-domain correlations, which restricts their generalization capability in complex scenarios.

## 3. Methods

### 3.1. Overall

Let the hyperspectral image (HS) be denoted as $X\in {\mathbb{R}}^{h\times w\times B}$, where h and w represent spatial dimensions while B signifies the number of spectral bands. Let the panchromatic image (PAN) be represented as $Y\in {\mathbb{R}}^{H\times W\times 1}$ with spatial resolutions H ≫ h and W ≫ w.

The spatial resolution of hyperspectral imagery is inherently constrained by sensor limitations, which can be formally modeled as

(1) $$X=D_{\mathrm{HS}}(Z)+N_{\mathrm{HS}}$$

where **Z** denotes the ideal high-spatial–spectral-resolution image (latent true scene) and $D_{\mathrm{HS}}$ represents the degradation operator of the hyperspectral sensor, comprising spatial blurring (point spread function effects) and downsampling (spectral and spatial resolution trade-off). $N_{\mathrm{HS}}\in {\mathbb{R}}^{h\times w\times B}$ signifies additive noise components in HS imagery.

The panchromatic imagery acquisition process, leveraging high-spatial-resolution sensors, can be mathematically formulated as

(2) $$Y=W_{\mathrm{PAN}}(Z)+N_{\mathrm{PAN}}$$

where $W_{\mathrm{PAN}}$ represents the spectral response function (SRF) operator that projects the spectral cube **Z** of the hyperspectral image onto a single-band panchromatic image. $N_{\mathrm{PAN}}\in {\mathbb{R}}^{H\times W\times 1}$ denote additive noise components in PAN imagery.

The objective of pansharpening is to generate a high-spatial–spectral-resolution fused image $Z\in {\mathbb{R}}^{H\times W\times B}$ through the synergistic integration of complementary information from **X** and **Y**.

(3) $$\underset{Z}{\min}\frac{1}{2}{\left\|X-D_{\mathrm{HS}}(Z)\right\|}_{F}^{2}+\frac{\mu }{2}{\left\|Y-W_{\mathrm{PAN}}(Z)\right\|}_{F}^{2}+\lambda \Phi (Z)$$

The first term enforces spectral consistency between the reconstructed image and the reference hyperspectral data, while the second term ensures spatial consistency with the panchromatic observation. The parameter *μ* controls the relative importance between spectral and spatial fidelity. The regularization term Φ(Z) encodes prior constraints on the solution, and *λ* is a weighting factor that balances the contribution of this prior.

Guided by the proposed bidirectional driving framework, we develop a novel end-to-end deep learning architecture, namely the Spatial–Spectral Bidirectional-Driven Collaborative Network (SSDCNet), to solve the aforementioned formulation.

### 3.2. Spatial–Spectral Bidirectional-Driven Collaborative Network

**Figure 1:**
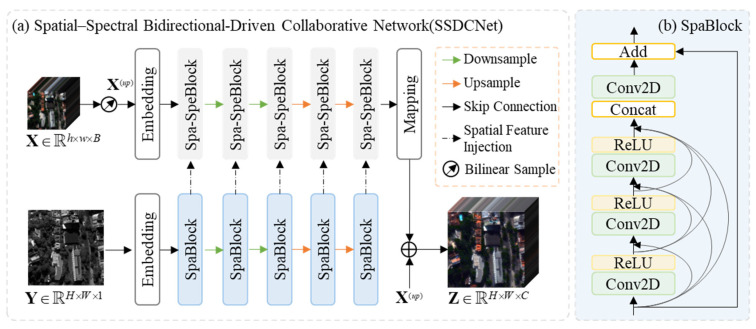
(**a**). Overall framework of the proposed SSDCNet. (**b**). Architecture of the SpaBlock.

As shown in Figure 1, the proposed SSDCNet adopts a novel asymmetric twin-branch architecture design, comprising a spectral-preserving main pathway and a spatial-enhanced parallel branch. The parallel branch employs a multi-level spatial feature extraction block (SpaBlock) to progressively distill hierarchical spatial details from the panchromatic imagery, implementing adaptive detail injection through spatial coordinates attention mechanisms.

(4) $$Y^{\left(i\right)}=\mathrm{SpaBlocks}(\mathrm{Embedding}(Y)),i=1,2,3,4,5$$

where Y^(*i*)^ denotes the output of the 5-level SpaBlock in Figure 1.

Concurrently, the main branch utilizes spectral self-attention mechanisms to maintain spectral integrity of the hyperspectral data while establishing bidirectional information flow with the parallel branch via a specially designed cross-modal interaction unit (Spa-SpeBlock).

(5) $$X^{\left(i\right)}=\mathrm{Spa}\text{-}\mathrm{SpeBlocks}(\mathrm{Embedding}(X^{\left(up\right)}),Y^{\left(i\right)}),i=1,2,3,4,5$$

where Y^(*i*)^ represents the output of the 5-level Spa-SpeBlock.

This dual-driven mechanism enables dynamic modulation between spatial enhancement and spectral preservation through explicit spatial–spectral interaction and feature recalibration modules, ultimately facilitating the reconstruction of high-quality hyperspectral images.

(6) $$Z=\mathrm{Mapping}(X^{\left(5\right)})+X^{\left(up\right)}$$

where **Z** denotes the final output of SSDCNet.

Both the Embedding and Mapping are implemented using 2D convolutional layers to establish bidirectional mappings between high-dimensional feature spaces and image domains.

### 3.3. Spatial Feature Extraction Block (SpaBlock)

As the primary module in the spatial pathway, SpaBlock is designed for dedicated spatial feature extraction and progressive refinement. It adopts a self-calibrated convolution-driven residual learning paradigm that integrates channel-wise feature recalibration via squeeze-and-excitation operations, spatial context aggregation through depth-expandable dilated convolutions, and cross-stage information fusion using dense skip connections. This design enables the module to capture both fine-grained textures and broader structural patterns in a hierarchical manner. By continuously refining spatial representations through residual error compensation, SpaBlock ensures the preservation of boundary sharpness and structural integrity, providing a stable and expressive spatial feature foundation for subsequent processing.

(7) $$x_{\left(i\right)}=Relu(\mathrm{Conv}2D(Concat(x_{in},x_{\left(1\right)},\ldots ,x_{\left(i-1\right)})))$$

(8) $$x_{out}=\mathrm{Conv}2D(Concat(x_{in},x_{\left(1\right)},\ldots ,x_{\left(i\right)}))+x_{in}$$

where *x_in_* and *x_out_* denote the input and output of the SpaBlock, respectively, and *x*_(*i*)_ represents the output of each densely connected layer. Concat denotes concatenation along the channel dimension, and ReLU is the activation function.

### 3.4. Cross-Modal Interaction Unit (Spa-SpeBlock)

**Figure 2:**
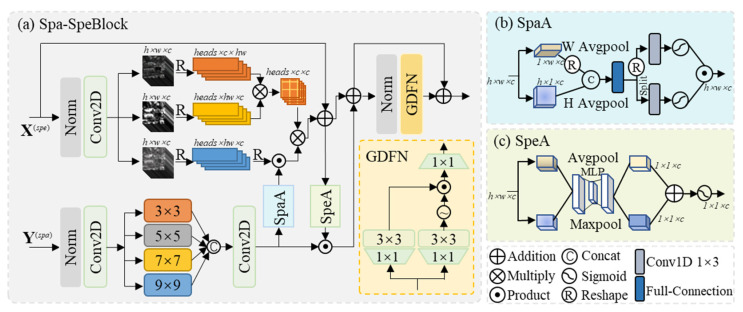
(**a**). Architecture of the Spa-SpeBlock, comprising spectral self-attention, multi-perspective spatial convolution, spatial feature coordinate weighting (SpaA), and spectral feature channel weighting (SpeA). (**b**). Architecture of the SpaA. (**c**). Architecture of the SpeA.

As the key component of the spatial–spectral interaction pathway, the Spa-SpeBlock (Figure 2) is designed to enable bidirectional coupling between spatial and spectral representations. Unlike SpaBlock, which focuses on intra-spatial feature extraction, Spa-SpeBlock emphasizes cross-dimensional interaction through a synergistic integration of spectral self-attention and multi-perspective spatial convolution. Specifically, the spectral branch leverages spatially guided attention to incorporate spatial context into spectral modeling, while the spatial branch employs parallel convolutions with varying receptive fields (e.g., 3 × 3, 5 × 5, 7 × 7, and 9 × 9) that are adaptively modulated by spectral responses. In this way, spatial features are no longer extracted independently, but are dynamically conditioned on spectral characteristics, enabling more effective alignment and fusion between the two domains.

In the spectral self-attention module, the input X^(*i*)^ is first normalized and then passed through depth-wise convolutions to generate the query (Q), key (K), and value (V) matrices, which are subsequently reshaped into vector forms.

(9) $$Q,K,V=split(\mathrm{Conv}2D(\mathrm{Norm}(X^{\left(i\right)})))$$

where *Split* represents split at the channel dimension.

Subsequently, the V vector is further refined through a spatial coordinate-guided attention reweighting W^(*spa*)^, which adaptively modulates spectral responses according to spatial saliency. In this way, coordinate-aware spatial information is implicitly embedded into spectral feature modeling, enabling the resulting spectral tokens to capture both inter-channel relationships and spatially varying structures.

(10) $$\overset{\text{\textasciitilde{}}}{V}=W^{\left(spa\right)}*V$$

The attention map is computed by performing a scaled dot-product operation between the Q and K vectors, followed by a softmax function to obtain the attention weights. These weights are then applied to the $\overset{\text{\textasciitilde{}}}{V}$ vector to generate the attended spectral features, which are finally reshaped back to their original dimensions.

(11) $$X^{\left(spe\right)}=softmax(Q^{T}K)\overset{\text{\textasciitilde{}}}{V}+X^{\left(i\right)}$$

This enables adaptive calibration of spectral features by emphasizing spatially salient regions while preserving inter-channel coherence.

In the multi-perspective spatial convolution module, the input Y^(*i*)^ is first normalized and then passed through parallel convolutional branches with varying receptive fields to extract spatial features under different contextual scopes, thereby enriching the feature representation. The outputs of these branches are subsequently concatenated and reshaped to restore the original spatial dimensions.

(12) $$Y^{\left(fea\right)}=\mathrm{Conv}2D(\mathrm{Norm}(Y^{\left(i\right)}))$$

(13) $$Y^{\left(spa\right)}=\mathrm{Conv}2D(Concat(3\times 3(Y^{\left(fea\right)}),5\times 5(Y^{\left(fea\right)}),7\times 7(Y^{\left(fea\right)}),9\times 9(Y^{\left(fea\right)})))$$

Subsequently, Y^(*spa*)^ is modulated by a set of spectral importance coefficients W^(*spe*)^ derived through spectral channel attention, which guides the spatial convolutional operations.

(14) $$Y^{\left(spa\right)}=W^{\left(spe\right)}*Y^{\left(spa\right)}$$

This modulation effectively mitigates spectral distortion while preserving spatial structural information.

### 3.5. Spatial Feature Coordinate Weighting (SpaA)

The SpaA module is designed to enhance the model’s perception of spatial structures by selectively emphasizing important spatial positions through targeted weighting across different spatial dimensions of the feature map.

Specifically, the input feature map is first reshaped along the width and height dimensions independently. Each reshaped feature map is then subjected to average pooling, which aggregates local spatial information to obtain representative statistics for each region. By applying average pooling along both the width and height axes, spatial statistical characteristics are captured across the two dimensions. The resulting pooled features from the width and height dimensions are concatenated along the channel dimension and passed through a fully connected layer.

(15) $$W=\mathrm{Linear}(Concat(\mathrm{AvgH}(Y^{\left(spa\right)}{)}^{T},\mathrm{AvgW}(Y^{\left(spa\right)})))$$

where AvgH and AvgW denote average pooling along the H and W directions, respectively.

The combined feature is then split into two branches, each processed by a separate 1D convolution and activation function. The outputs from the two branches are then multiplied via matrix multiplication to generate the final spatial weighting coefficients W^(*spa*)^.

(16) $$W^{\left(spa\right)}=(Relu(\mathrm{Conv}1D(W^{T})))(Relu(\mathrm{Conv}1D(W)))$$

### 3.6. Spectral Feature Channel Weighting (SpeA)

The SpeA module focuses on enhancing the model’s ability to discriminate and utilize spectral features by emphasizing channels that carry significant spectral information.

Initially, the input feature map undergoes both average pooling and max pooling operations along the spatial dimensions. The average pooling aggregates the spatial information within each channel to generate a representative value, while max pooling captures the most prominent local features, providing complementary information to the average pooling results. The resulting feature vectors from both pooling operations are then fed into a multi-layer perceptron (MLP), which models the inter-channel relationships and assesses the importance of each channel. The two outputs are summed and passed through an activation function to produce the final spectral channel weighting coefficients.

(17) $$W^{\left(spe\right)}=Relu(\mathrm{MLP}(\mathrm{Avg}(X^{\left(spe\right)}))+\mathrm{MLP}(\mathrm{Max}(X^{\left(spe\right)})))$$

where avg denotes average pooling and max denotes max pooling.

## 4. Experiments

To validate the performance of SSDCNet, we conduct two types of experiments: simulated experiments and real-world experiments.

### 4.1. Datasets

In the simulated setting, we utilize three publicly available high-resolution hyperspectral datasets: WorldView-3, Chikusei, and ZY-1-02D. For real-world evaluation, we adopt the ZY-1-02D dataset acquired by the Ziyuan-1-02D satellite.

(a) WorldView-3: The panchromatic image has a spatial resolution of 0.3 m, while the multispectral image has a spatial resolution of 1.2 m. The LR-HSI is processed into a size of 16 × 16 × 8, the HR-PAN into 64 × 64 × 1, and the ground-truth (GT) HR-HSI into 64 × 64 × 8, all derived using the method proposed in [37]. The training set contains 9714 samples, while the test set includes 1080 samples.
(b) Chikusei: The dataset has a ground sampling distance of 2.5 m. The LR-HSI is processed into 16 × 16 × 128, the HR-PAN into 64 × 64 × 1, and the GT into 64 × 64 × 128, following the approach described in [35]. The original image size is 2335 × 2517. A 512 × 512 patch is selected for testing, and the remaining regions are used for training.
(c) ZY-1-02D: The hyperspectral image has a spatial resolution of 30 m, while the panchromatic image has a spatial resolution of 2.5 m. In the simulated experiment, the LR-HSI is processed into 10 × 10 × 76, the HR-PAN into 120 × 120 × 1, and the GT HR-HSI into 120 × 120 × 76. The original GT image has a resolution of 1485 × 1344; a 360 × 360 patch is used for testing, and the rest is used for training. In the real-world experiment, the PAN image has a resolution of 17,820 × 16,128, and the entire image is used for testing.

### 4.2. Experiments Details

In the simulated experiments, SSDCNet was trained for 300 epochs, with each epoch consisting of 100 iterations. A batch size of 10 was used in each iteration. The HR-HSI served as the GT. The LR-HSI was generated by applying Gaussian blurring followed by spatial downsampling, while the HR-PAN was synthesized using a spectral response function.

During training, the model is optimized in a supervised manner using an L1 reconstruction loss between the predicted high-resolution hyperspectral image **Z** and the ground truth:

(18) $$L=\|Z,\mathrm{GT}{\|}_{1}$$

This loss can be viewed as a practical surrogate of the general formulation in Equation (3), where the network implicitly learns to satisfy both spatial and spectral consistency constraints through data-driven optimization, rather than explicitly solving each term in the formulation.

The model was optimized using the Adam optimizer with a learning rate of 2 × 10^−4^.

In the real-world experiments, the model was trained following Wald’s protocol for quantitative evaluation. A total of 100 epochs were conducted, with each epoch consisting of 100 iterations. A batch size of 10 was used in each iteration. The training was performed using the Adam optimizer with a learning rate of 2 × 10^−4^.

We compare our proposed SSDCNet with seven state-of-the-art hyperspectral image fusion algorithms: FusionNet (2021) [19], GPPNN (2021) [38], Panformer (2022) [29], PMACNet (2022) [36], LGPConv (2023) [39], DISPNet (2024) [40], and GGPNet (2025) [41]. To comprehensively evaluate the performance of all methods, we employ the following commonly used quantitative metrics [42]: Peak Signal-to-Noise Ratio (PSNR), Structural Similarity Index (SSIM), Spectral Angle Mapper (SAM), Root Mean Square Error (RMSE), and Erreur Relative Globale Adimensionnelle de Synthèse (ERGAS).

To ensure a fair comparison, all baseline methods and our proposed approach were uniformly retrained and tested on the same server platform, thereby eliminating potential biases due to hardware variations. The experiments were conducted on a server equipped with an NVIDIA Tesla V100 GPU and running the Ubuntu 18.04.6 LTS operating system.

### 4.3. Simulated Experiments Results

**Figure 3:**
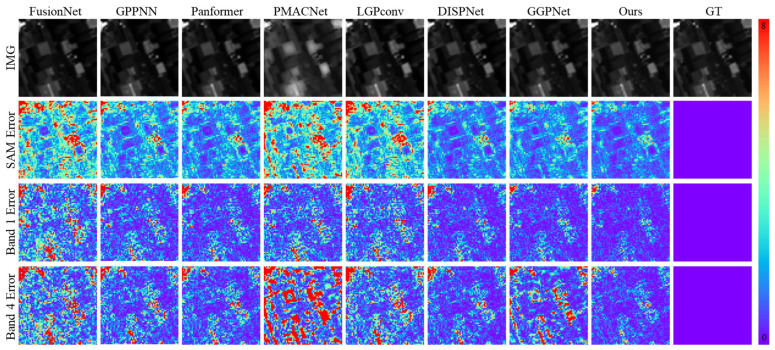
Visual Comparison of Reconstruction Results and Error Maps Across Different Methods on the Simulated WorldView-3 Dataset.

As shown in Figure 3, in the visual comparison on the WorldView-3 dataset, the proposed method demonstrates significant advantages in both spectral feature capture and spatial detail reconstruction. The SAM maps indicate that our method produces substantially fewer high-angular-error regions compared to methods such as FusionNet, PMACNet, and LGPConv, with the high-error areas being more spatially concentrated.

Quantitative analysis in Table 1 shows that our method achieves a SAM value of 3.2663°, representing a 3.0% reduction compared to the second-best method, DISPNet (3.3677°), and a 10.6% reduction relative to FusionNet (3.6513°), indicating superior spectral fitting accuracy and an enhanced ability to reduce angular deviations between predicted and ground-truth spectra. In contrast, FusionNet and PMACNet produce SAM maps with widespread high-angular-error regions, reflecting insufficient sensitivity to local spectral features during multispectral information fusion. Although Panformer and DISPNet improve spectral consistency in certain bands via attention mechanisms, their overall spectral angular errors remain higher than those of the proposed method.

In addition to reconstruction accuracy, we further analyze computational efficiency in terms of inference time and memory consumption. As shown in Table 1, attention-based methods such as Panformer and GGPNet incur noticeable computational overhead, with inference times of 14.889 ms and 21.593 ms, respectively. While DISPNet achieves competitive accuracy, it relies on a relatively large model size (7.17 M parameters). In comparison, our method attains the best overall reconstruction performance while maintaining a moderate model complexity (1.14 M parameters and 1.01 G FLOPs), demonstrating a more favorable trade-off between accuracy and efficiency. Although the peak memory usage is higher than that of lightweight models, the proposed method avoids the excessive computational burden associated with heavy attention designs and achieves competitive inference speed (11.863 ms), thereby validating the effectiveness of our architecture in balancing performance and practical efficiency.

**Figure 4:**
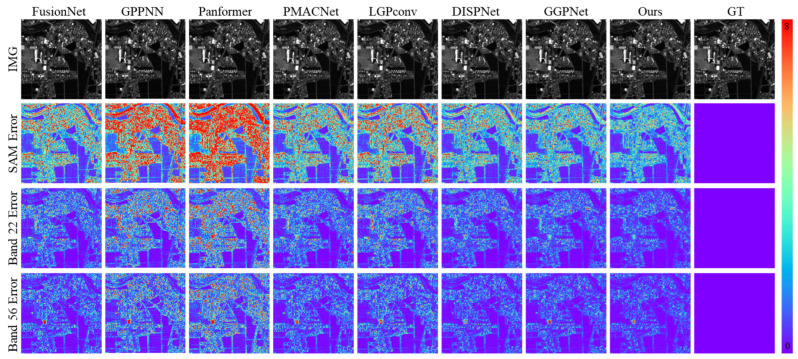
Visual Comparison of Reconstruction Results and Error Maps Across Different Methods on the Simulated Chikusei Dataset.

As validated by the results in Figure 4, the visual results on the Chikusei dataset further confirm the spectral fidelity and error control capability of the proposed method. The SAM error maps reveal that GPPNN and Panformer exhibit significant angular deviations in low-reflectance areas such as urban regions and water bodies. As indicated by the quantitative analysis in Table 2, their SAM values reach 3.5122° and 3.5971°, respectively, reflecting insufficient adaptability in spatial–spectral feature fusion. Although PMACNet and DISPNet alleviate part of the spectral confusion through local feature learning, they still struggle to prevent spectral distortion under high-spectral-resolution demands, with SAM values of 3.1441° and 2.9324°, both higher than that of our method (2.7833°). In contrast, our approach significantly reduces the spatial extent of high-angular-error regions through dynamic spectral modulation, achieving a 5.1% improvement over DISPNet and a 20.7% reduction compared to GPPNN, thereby demonstrating enhanced spectral representation capability.

Error map analysis further shows that, in the pan-sharpening task, the proposed method yields error distributions more closely aligned with actual object boundaries. The RMSE (0.0059) and ERGAS (3.2475) are reduced by 24.4% and 7.3%, respectively, compared to GPPNN, while GPPNN and Panformer exhibit higher error values in similar heterogeneous regions, indicating limitations in their spatial–spectral interaction modeling. Additionally, our method achieves a PSNR of 45.09 dB and an SSIM of 0.9918, representing average improvements of 1.2% and 0.2% over competing methods. In particular, in regions such as building edges and vegetation boundaries, the proportion of high-error areas in the error heatmaps is reduced to less than 0.3%, significantly outperforming DISPNet (0.8%).

Beyond reconstruction accuracy, we further analyze computational efficiency. As shown in Table 2, attention-intensive models such as GGPNet and Panformer exhibit substantial computational overhead, with inference times of 33.779 ms and 14.797 ms, respectively, as well as significantly increased memory consumption. While DISPNet achieves competitive spectral accuracy, it relies on a considerably larger model size (14.33 M parameters and 55.94 G FLOPs). In contrast, the proposed method attains the best overall reconstruction performance while maintaining a moderate computational complexity (18.80 G FLOPs) and competitive inference time (11.907 ms). Although the peak memory usage is relatively high, the proposed design avoids excessive reliance on heavy attention mechanisms and achieves a more balanced trade-off between accuracy and efficiency, further validating the practicality of the proposed architecture.

**Figure 5:**
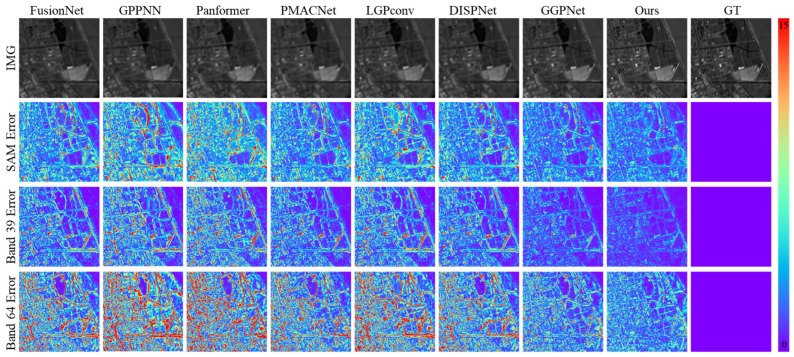
Visual Comparison of Reconstruction Results and Error Maps Across Different Methods on the Simulated ZY-1-02D Dataset.

Figure 5 highlights that, under complex land-cover scenes in the ZY-1-02D dataset, the proposed method achieves notable improvements in both spectral accuracy and spatial detail reconstruction. Analysis of the SAM maps reveals that our method attains a SAM value of 1.6776°, representing a 6.8% reduction compared to the second-best method PMACNet (1.7994°), and a 17.2% reduction compared to FusionNet (2.0270°). The proportion of high-angular-error regions is significantly lower than that observed in methods such as FusionNet, GPPNN, and Panformer, indicating the superior capability of our method in capturing spectral features. The error maps further show that, for fine-scale objects (e.g., road markings and vehicle contours), the RMSE achieved by our method (0.0068) is 43.8% lower than that of LGPConv (0.0121), with fewer high-error areas and an error distribution more closely aligned with actual object boundaries, validating its precision in sub-pixel detail reconstruction.

Quantitative results in Table 3 further demonstrate that our method achieves a PSNR of 44.17 dB and an SSIM of 0.9832, reflecting average improvements of 9.7% and 2.4%, respectively, over competing methods. In texture-dense regions, the proportion of high-error areas in the error heatmaps (>5% error) is below 0.1%, significantly outperforming DISPNet’s 0.5%. Moreover, our method yields an ERGAS value of 0.5732, which is 42.2% lower than that of GPPNN (0.9912), indicating a clear advantage in maintaining global spectral consistency. In contrast, LGPConv shows a wider error distribution in similar regions (SAM = 2.1760°, RMSE = 0.0121), highlighting the limitations of fixed receptive field convolutions in adapting to scale variations inherent in high-resolution remote sensing imagery.

In addition to reconstruction performance, we further evaluate computational efficiency. As shown in Table 3, attention-heavy models such as GGPNet and Panformer incur substantial computational overhead, with high FLOPs (163.41 G and 6.11 G, respectively) and increased inference time (41.007 ms and 15.393 ms). Although GGPNet achieves competitive accuracy, it does so at the cost of significantly higher computational complexity and memory consumption (297.05 MB). Similarly, PMACNet and DISPNet rely on relatively large models and exhibit slower inference speeds. In comparison, the proposed method achieves the best overall reconstruction performance while maintaining a moderate computational cost (25.90 G FLOPs) and a reasonable inference time (16.460 ms). Although the peak memory usage is relatively high, the proposed architecture avoids excessive reliance on heavy attention operations and achieves a more balanced trade-off between accuracy and efficiency, further demonstrating its practicality for real-world applications.

### 4.4. Real-World Experiments Results

To evaluate the practical utility of the proposed method in complex orbital environments, we constructed a large-scale real-world benchmark dataset using imagery from the ZY-1-02D satellite. This dataset presents a significant challenge for pansharpening due to the large 12× spatial resolution gap between the 2.5 m panchromatic band and the 30 m hyperspectral sensor (comprising 76 spectral bands).

To ensure high-fidelity inputs, a rigorous preprocessing pipeline was implemented. First, raw digital numbers were converted to top-of-atmosphere reflectance and subsequently processed using the FLAASH (Fast Line-of-sight Atmospheric Analysis of Spectral Hypercubes) model for atmospheric correction to eliminate scattering and absorption effects. Second, geometric orthorectification was performed using Rational Polynomial Coefficients (RPCs) and a high-resolution Digital Elevation Model (DEM) to correct terrain-induced distortions. Finally, an intensity-based sub-pixel registration strategy was employed to ensure precise spatial–spectral alignment across the scene.

After preprocessing, the dataset was further prepared for model training and evaluation. Specifically, the large-scale scene (PAN: 17,820 × 16,128; HSI: 1485 × 1344) was divided into non-overlapping patches. For training, image patches of size 120 × 120 (PAN) and the corresponding 10 × 10 hyperspectral patches were extracted, following the degradation protocol used in simulated experiments. A fixed region of size 360 × 360 (PAN domain) was reserved for testing to ensure consistency across comparisons, while the remaining regions were used for training.

All hyperspectral bands were normalized to [0, 1] prior to training. In addition, bands with severe noise at spectral extremes were removed to improve data quality. This patch-based construction not only facilitates efficient model training but also ensures that both global structure and local details are adequately represented. To facilitate community-wide research on real-world pansharpening, the complete dataset has been made publicly available on Google.

**Figure 6:**
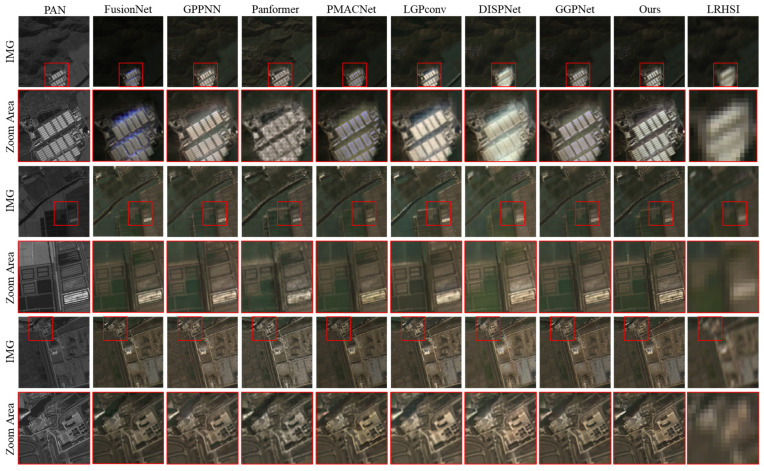
Visual Comparison of Reconstruction Results Across Different Methods on the Real-World ZY-1-02D Dataset.

As illustrated in Figure 6, the real-world fusion results on the ZY-1-02D dataset further validate the superiority of the proposed method in handling complex and heterogeneous remote sensing scenes. Compared with baseline methods including FusionNet, GPPNN, Panformer, PMACNet, LGPConv, DISPNet, and GGPNet, our method demonstrates more faithful spectral reconstruction and clearer spatial structures.

In the first and second zoomed-in regions, which contain dense industrial buildings and roof structures, our method preserves edge contours and fine geometric details more accurately, while other methods either suffer from spectral distortion (e.g., false coloring in FusionNet and Panformer) or over-smoothing effects (as observed in PMACNet, GGPNet and LGPConv). DISPNet and GPPNN exhibit visible structure artifacts or lack clarity in high-frequency details, especially around the boundary regions of rooftops and narrow roads.

In the third zoom-in region, which includes narrow linear features and contours, the proposed method shows a better reconstruction of sub-pixel structures, achieving superior spatial fidelity without introducing significant spectral artifacts. Other methods either blur the contours (e.g., LGPConv and PMACNet) or introduce ghosting effects due to poor alignment of spatial and spectral cues (e.g., FusionNet).

These observations underscore the generalization ability of our model to real satellite data without relying on ground-truth HR-HSI supervision.

### 4.5. Ablation Studies

To systematically validate the effectiveness of the proposed spatial–spectral interaction mechanism from both architectural design and feature modulation perspectives, we conduct two complementary ablation studies focusing on structural coupling and interaction-driven attention modeling, respectively.

First, to further validate the effectiveness of the proposed spatial–spectral interaction mechanism, we conduct an additional ablation study by replacing our unified bidirectional design with a conventional decoupled dual-branch architecture. Specifically, in this variant, spatial and spectral features are extracted independently in two separate branches, and additional interaction modules are inserted between them to compensate for the lack of intrinsic coupling. This design mimics typical dual-path strategies where spatial and spectral representations are processed in isolation and only partially fused through explicit operations.

The comparison results are reported in Table 4. It can be observed that the decoupled dual-branch variant achieves comparable reconstruction performance in terms of PSNR, SSIM, and SAM. However, this comes at the cost of significantly increased model complexity, with parameter count and computational overhead rising from 13.59 M to 19.55 M and from 25.90 G to 36.23 G FLOPs, respectively. This indicates that simply introducing additional interaction modules on top of separated branches leads to redundant representations and inefficient computation.

In contrast, the proposed framework embeds spatial–spectral interaction directly within the feature extraction process by treating the spectral pathway as an inherent carrier of cross-dimensional information. This design avoids artificial separation between spatial and spectral modeling, enabling more efficient and compact representation learning.

Second, to demonstrate the effectiveness of the proposed bidirectional spatial–spectral driving mechanism, we conduct a comprehensive ablation study by jointly evaluating conventional attention strategies and the proposed interaction modules within a unified framework. Specifically, we consider multiple model variants, including the baseline without any attention modules, variants equipped with conventional spatial attention (SA), implemented via sigmoid-based weighting along the spatial dimension, and channel attention (CA), implemented via sigmoid-based weighting along the channel dimension, and variants incorporating the proposed spatial feature coordinate weighting module (SpaA) and spectral feature channel weighting module (SpeA), either individually or jointly.

As shown in Table 5, the baseline model exhibits the lowest performance across all evaluation metrics, indicating its limited capability in capturing fine-grained spatial and spectral details. Introducing conventional attention mechanisms (SA or CA) leads to only marginal improvements. While SA enhances spatial representation through spatial-wise reweighting and CA improves spectral consistency via channel-wise modulation, both approaches operate strictly within their respective dimensions and fail to capture the intrinsic coupling between spatial structures and spectral responses.

In contrast, the proposed SpaA and SpeA modules bring more substantial and consistent improvements. When applied individually, SpaA improves spatial structure perception through coordinate-aware weighting, while SpeA enhances spectral fidelity, as reflected by the reduction in SAM and ERGAS. More importantly, when both modules are jointly integrated, the performance is further boosted across all metrics, suggesting that the interaction between spatial and spectral domains is being more effectively exploited during feature modeling.

A direct comparison between conventional attention (SA/CA) and the proposed modules (SpaA/SpeA) under the same setting reveals that simple intra-dimensional recalibration is insufficient to fully leverage cross-dimensional dependencies. Instead, the proposed design enables a more structured and effective form of interaction, where spatial cues guide spectral responses and spectral characteristics in turn refine spatial representations.

## 5. Conclusions

This work presents a unified bidirectional driving framework for hyperspectral pansharpening, addressing long-standing challenges in balancing spatial and spectral fidelity. By dynamically injecting coordinate-aware spatial features into the spectral self-attention process and employing spectral importance scores to modulate spatial convolutions through spectral channel weighting, our method establishes a cross-dimensional interaction that effectively reduces spectral distortion while enhancing spatial sharpness. Unlike conventional dual-branch architectures or constrained attention mechanisms, our design enables continuous and reciprocal guidance between spatial and spectral domains. Extensive quantitative and qualitative evaluations on both simulated datasets and real-world ZY-1-02D imagery confirm the robustness and effectiveness of our approach, achieving superior reconstruction quality under complex remote sensing conditions.

## Figures and Tables

**Table 1:** Quantitative Comparison of Different Methods on the Simulated WorldView-3 Dataset (bold values indicate the best performance for each metric).

Model	PSNR	SSIM	SAM	RMSE	ERGAS	Paras (M)	Flops (G)	Inference Time (ms)	Peak Memory (MB)
FusionNet [19]	37.31	0.9673	3.6513	0.0173	2.6069	1.14	0.15	0.805	14.70
GPPNN [38]	37.71	0.9695	3.5476	0.0166	2.5407	0.24	0.65	4.130	130.47
Panformer [29]	37.98	0.9710	3.5096	0.0163	2.4320	1.36	0.64	14.889	19.91
PMACNet [36]	37.32	0.9607	3.9328	0.0206	3.5799	0.95	3.59	2.074	144.28
LGPconv [39]	37.30	0.9668	3.8018	0.0176	2.6441	0.13	0.05	1.472	50.43
DISPNet [40]	38.31	0.9726	3.3677	0.0157	2.3431	7.17	1.76	10.260	14.20
GGPNet [41]	38.10	0.9715	3.3905	0.0164	2.3823	3.26	30.85	21.593	169.71
Ours	**38.83**	**0.9757**	**3.2663**	**0.0148**	**2.1984**	1.14	1.01	11.863	149.71

**Table 2:** Quantitative Comparison of Different Methods on the Simulated Chikusei Dataset (bold values indicate the best performance for each metric).

Model	PSNR	SSIM	SAM	RMSE	ERGAS	Paras (M)	Flops (G)	Inference Time (ms)	Peak Memory (MB)
FusionNet [19]	43.86	0.9892	3.1805	0.0068	3.6623	5.02	19.69	0.981	45.78
GPPNN [38]	42.75	0.9857	3.5122	0.0078	3.5047	7.60	20.57	5.782	169.18
Panformer [29]	42.42	0.9854	3.5971	0.0079	4.2124	5.24	2.96	14.797	54.03
PMACNet [36]	43.86	0.9891	3.1441	0.0068	3.6475	6.63	16.85	4.818	68.98
LGPconv [39]	43.57	0.9881	3.2317	0.0071	3.8167	2.59	1.43	1.433	155.90
DISPNet [40]	44.53	0.9907	2.9324	0.0063	3.4502	14.33	55.94	10.121	123.52
GGPNet [41]	44.60	0.9908	2.9220	0.0063	3.4041	13.80	131.83	33.779	277.32
Ours	**45.09**	**0.9918**	**2.7833**	**0.0059**	**3.2475**	17.84	18.80	11.907	251.44

**Table 3:** Quantitative Comparison of Different Methods on the Simulated ZY-1-02D Dataset (bold values indicate the best performance for each metric).

Model	PSNR	SSIM	SAM	RMSE	ERGAS	Paras (M)	Flops (G)	Inference Time (ms)	Peak Memory (MB)
FusionNet [19]	39.60	0.9480	2.0270	0.0114	0.9623	10.03	66.33	2.706	124.24
GPPNN [38]	39.46	0.9458	2.1276	0.0117	0.9912	2.68	24.88	6.941	163.51
Panformer [29]	39.93	0.9511	2.0792	0.0107	0.9124	9.63	6.11	15.393	137.14
PMACNet [36]	40.27	0.9556	1.7994	0.0104	0.8798	11.53	54.73	12.828	279.44
LGPconv [39]	39.08	0.9409	2.1760	0.0121	1.0212	7.58	1.82	2.239	190.75
DISPNet [40]	39.61	0.9478	1.8651	0.0113	0.9549	4.85	63.05	12.118	161.76
GGPNet [41]	42.75	0.9708	1.7331	0.0088	0.8004	4.86	163.41	41.007	297.05
Ours	**44.17**	**0.9832**	**1.6776**	**0.0068**	**0.5732**	13.59	25.90	16.460	269.74

**Table 4:** Comparison between the proposed unified spatial–spectral interaction framework and a decoupled dual-branch variant with explicit intermediate interactions, in terms of reconstruction performance and computational efficiency.

	PSNR	SSIM	SAM	RMSE	ERGAS	Paras	Flops
Ours	44.17	0.9832	1.6776	0.0068	0.5732	13.59	25.90
Decoupled spatial–spectral	44.20	0.9838	1.6763	0.0068	0.5705	19.55	36.23

**Table 5:** Ablation study on the effectiveness of the bidirectional spatial–spectral interaction mechanism, comparing the proposed modules (SpaA, SpeA) with conventional spatial and channel attention (SA, CA).

Model	SpaA	SpeA	PSNR	SSIM	SAM	RMSE	ERGAS
Baseline	✗	✗	43.57	0.9781	1.8425	0.0074	0.6254
Baseline + SA	✗	✗	43.60	0.9781	1.8422	0.0073	0.6192
Baseline + SpaA	✓	✗	43.89	0.9802	1.8213	0.0070	0.5971
Baseline + CA	✗	✗	43.69	0.9790	1.7956	0.0073	0.6105
Baseline + SpeA	✗	✓	43.76	0.9795	1.7677	0.0071	0.5823
SpaA + SpeA	✓	✓	44.17	0.9832	1.6776	0.0068	0.5732

## Data Availability

The data are available: https://drive.google.com/drive/folders/1x4aQhkNKrQFRqqowT1aBB0bIMTjqSsN_?usp=sharing (accessed on 1 January 2026).
